# Overexpression of a MYB Family Gene, *OsMYB6*, Increases Drought and Salinity Stress Tolerance in Transgenic Rice

**DOI:** 10.3389/fpls.2019.00168

**Published:** 2019-02-18

**Authors:** Yuehui Tang, Xinxin Bao, Yuling Zhi, Qian Wu, Yaru Guo, Xuhui Yin, Liqin Zeng, Jia Li, Jing Zhang, Wenlong He, Weihao Liu, Qingwei Wang, Chengkai Jia, Zhengkang Li, Kun Liu

**Affiliations:** ^1^Key Laboratory of Plant Genetics and Molecular Breeding, Zhoukou Normal University, Zhoukou, China; ^2^Henan Key Laboratory of Crop Molecular Breeding and Bioreactor, Zhoukou, China; ^3^School of Journalism and Communication, Zhoukou Normal University, Zhoukou, China; ^4^College of Life Science and Agronomy, Zhoukou Normal University, Zhoukou, China

**Keywords:** MYB transcription factor, *OsMYB6*, drought, salt, rice

## Abstract

MYB transcription factors have been demonstrated to play key regulatory roles in plant growth, development and abiotic stress response. However, knowledge concerning the involvement of rice *MYB* genes in salinity and drought stress resistance are largely unknown. In the present study, we cloned and characterized the *OsMYB6* gene, which was induced by drought and salinity stress. Subcellular localization of OsMYB6-YFP fusion protein in protoplast cells indicated that OsMYB6 was localized in the nucleus. Overexpression of *OsMYB6* in rice did not suggest a negative effect on the growth and development of transgenic plants, but *OsMYB6*-overexpressing plants showed increased tolerance to drought and salt stress compared with wild-type plants, as are evaluated by higher proline content, higher CAT and SOD activities, lower REL and MDA content in transgenic plants under drought and salt stress conditions. In addition, the expression of abiotic stress-responsive genes were significantly higher in *OsMYB6* transgenic plants than that in wild-type plants under drought and salt stress conditions. These results indicate that *OsMYB6* gene functions as a stress-responsive transcription factor which plays a positive regulatory role in response to drought and salt stress resistance, and may be used as a candidate gene for molecular breeding of salt-tolerant and drought-tolerant crop varieties.

## Introduction

High salinity and drought are two major stress factors that can seriously affect plant growth, development, and crop yield. As sessile organisms, in the long-term evolution process, plants have evolved extraordinary and intricate defense mechanisms to better adapt to these extreme environmental conditions. When plants are exposed to drought and salinity stress, a series of genes are activated or inhibited, and the products of these genes may either further control the expression of downstream genes or directly protect plants from stress damage (Dai et al., 2007). Among these genes, transcription factors, such as AP2/ERF, MYB, NAC, WRKY, bZIP, and bHLH, have been demonstrated to play an important regulatory role in plant response to abiotic stress (Butt et al., 2017; Mao et al., 2017; Tang et al., 2017; Wu et al., 2017; Sun et al., 2018; Zhu et al., 2018).

The MYB transcription factor contains a MYB domain that is highly conserved across all eukaryotes and is located at the N terminus, whereas the C terminus is variable, acting as a trans-acting domain, involves in the regulation of a wide range of functions in the MYB protein (Butt et al., 2017). In addition, MYB proteins contain one, two or three imperfect repeats containing 52 amino acid residues in their MYB domain (Stracke et al., 2001; Butt et al., 2017). Based on the number of repeats in their MYB domain, the members from MYB family are divided into four groups, R2R3-MYB, MYB1-R, 4R-MYB, and R1R2R3-MYB in *Arabidopsis* (Stracke et al., 2001).

Since the first plant protein containing the MYB domain, named *c1*-encoded protein, is isolated in maize (Pazares et al., 1987), numerous MYB proteins have been identified in various plant species (e.g., rice, soybean, *Arabidopsis*, sweet orange) through genome-wide analysis method (Stracke et al., 2001; Chen et al., 2006; Du et al., 2012; Liu C. et al., 2014). Subsequent researches show that MYB transcription factor plays a significant role in regulatory networks that involve in the whole process of plant growth and development. For example, overexpression of *MusaMYB31* reduces total polyphenols content in banana (Tak et al., 2017). *DcMYB6* is a potentially key regulator involved in the regulation of anthocyanin biosynthesis in purple carrots (Xu et al., 2017). *EsMYB9* participates in modulating the flavonoid biosynthetic pathway in Epimedium (Huang et al., 2017). Furthermore, numerous studies have suggested that MYB proteins are also involved in regulating plant responses to abiotic stress (Lv et al., 2017; Wang et al., 2017; Wei et al., 2017). Overexpression of *TaODORANT1* enhances resistance to drought and salt stress in transgenic plants (Wei et al., 2017). *PbrMYB21* gene plays a positive role in drought tolerance partly due to the modulation of polyamine synthesis by regulating the ADC expression (Li et al., 2017). The CaMYB85 protein confers drought and salt tolerance via increasing the expression of stress-responsive genes in transgenic *Arabidopsis* (Butt et al., 2017). Taken together, although many MYB transcription factors have been extensively cloned and functionally analyzed, these abiotic stress-responsive MYB proteins remain relatively poorly characterized in rice.

Rice (*Oryza sativa*) is a monocotyledonous model plant and a staple crop for most of the world’s population. However, rice is more sensitive to drought and salt stress than other cereals such as barely, wheat, and rye (Huang et al., 2014). Therefore, identifying the abiotic stress-responsive *MYB* genes from rice plants and studying their molecular mechanism will enrich our understanding of the stress signal network in rich and be important to improve rice resistance to abiotic stress. In previous research, we notice that a MYB transcription factor, we named *OsMYB6*, which is strongly induced by drought and salt stress (Huang et al., 2014; Zhou et al., 2016). In addition, the homologous genes of *OsMYB6* gene in other species have not been studied and functionally characterized. The *OsMYB6* gene function study can provide a valuable reference for the study of homologous gene function in other plants. Thus, *OsMYB6* gene is selected for subsequent functional analysis. In this study, firstly, we isolated the *OsMYB6* gene from rice. Secondly, we tested the expression pattern of *OsMYB6* in different tissues, as well as exposed to drought and salt. Finally, we examined the role of the *OsMYB6* gene in tolerance to drought and salt stress by overexpressing the *OsMYB6* in rice. The work not only provides valuable information for exploring the role of the *MYB* genes in response to abiotic stress in rice, but also provides a candidate gene in molecular breeding to increase crop drought and salt stress resistance.

## Materials and Methods

### Plant Materials

ZH11 (*Oryza sativa* L.) was used as wild-type for analyses of *OsMYB6* gene expression in different tissues, as well as exposed to abiotic stress. For the analysis of *OsMYB6* gene expression profiles in rice, the roots and leaves of seedlings at the five-leaf stage, stems, panicles and seeds of 7 days after pollination at natural light were sampled and stored at -80°C. For salt stress treatment, 2-week-old seedlings of rice were cultivated in Yoshida’s culture solution containing 150 mM NaCl at 25°C under 16 h light/8 h dark in a temperature-controlled growth room. For drought stress treatment, 2-week-old seedlings of rice were cultivated in Yoshida’s culture solution with 20% PEG6000 at 25°C under 16 h light/8 h dark in a temperature-controlled growth room. For cold stress treatment, 2-week-old seedlings of rice were cultivated in Yoshida’s culture solution at 4°C under 16 h light/8 h dark. For GA_3_ stress treatment, 2-week-old seedlings of rice were cultivated in Yoshida’s culture solution with 10 μM GA_3_ at 25°C under 16 h light/8 h dark. For ABA stress treatment, 2-week-old seedlings of rice were cultivated in Yoshida’s culture solution with 50 μM ABA at 25°C under 16 h light/8 h dark. For H_2_O treatment, 2-week-old seedlings of rice were cultivated in Yoshida’s culture solution under 16 h light/8 h dark. The third Leaf samples were collected at 0 h, 3 h, 6 h, and 12 h of H_2_O, drought, salt, cold, ABA, and GA_3_ stresses and stored at -80°C. All of the experiments contained three biological replicates.

### Bioinformatics Analysis of *OsMYB6* Gene

The OsMYB6 protein and its homologous protein sequences were downloaded from the National Center for Biotechnology Information (NCBI^1^). DNAMAN software was used to analyze the amino acid sequences of *OsMYB6* and other MYB transcription factors. ExPASy^2^ was used to analysis the physical and chemical parameters for OsMYB6 protein.

### Subcellular Localization of OsMYB6 Protein

The coding sequence of *OsMYB6* gene without stop codon was amplified through RT-PCR with 2 μL cDNA from roots and leaves. Then the PCR products was ligated with *EcoR* I and *Kpn* I-digested pSAT6-eYFP-N1 vector to generate pSAT6-OsMYB6-YFP fusion expression vector derived by CaMV 35S (cauliflower mosaic virus 35S) promoter. The constructed OsMYB6-YFP fusion expression vector was confirmed by sequencing. Subsequently, this fusion vector and the control vector (pSAT6-eYFP-N1) were transformed into *Arabidopsis* protoplast cells by PEG-mediated method, respectively. YFP fluorescence in transformed *Arabidopsis* protoplast cells was detected under a laser scanning confocal microscope. *Arabidopsis* protoplast cells were prepared based on a previous report (Hoffman et al., 1994).

### Gene Cloning and Generation of Transgenic Rice of *OsMYB6* Gene

The full-length cDNA of *OsMYB6* were amplified with the specific primers by RT-PCR using 2 μL cDNA from roots and leaves. The product was connected to the pMD18-T vector (TAKARA, Beijing, China) and sequenced. Then the correct *OsMYB6* sequence digested from pMD18-T-*OsMYB6* vector was cloned into the *Kpn* I-*Xba* I sites of the pCAMBIA1301 vector under the control of CaMV 35S promoter. Furthermore, the pCAMBIA1301 vector also contained a β-glucuronidase (GUS) gene under the control of the CaMV 35S promoter. The pCAMBIA1301-*OsMYB6* construct was transformed into *Agrobacterium tumefaciens* EHA105 by the freeze–thaw procedure, and then the EHA105-meditated methods were used to transform the 35S::*OsMYB6* expression construct into rice embryonic calli (Tang et al., 2017). Transgenic lines were verified by hygromycin screening and GUS staining. GUS staining was preformed according to previous report (Ye et al., 2017). In addition, the hygromycin-resistant plants were further confirmed by PCR method using specific primer. Finally, qRT-PCR was used to further identify efficient transgenic plants by detecting the expression of *OsMYB6* gene in wild-type lines and transgenic lines. Homozygous T3 progeny transgenic lines were used for subsequent experimental analysis.

### Pollen Germination Assays and Phenotype Analysis

Pollen from transgenic plants with *OsMYB6* gene and wild-type plants at flowering time was used for pollen germination analysis. Pollen germination were determined according to the methods described by Shi et al. (2018). Olympus BX-URA2 microscope (Japan) was used to observe pollen germination. Furthermore, a total of 30 individual plants each of the transgenic (OE1, OE2, and OE3) and wild-type plants were used to analyze the seed setting rate, pollen fertility, 1,000-seed weight and yield per plant.

### Stress Tolerance of Transgenic Rice With *OsMYB6* Gene

For drought stress treatment, 2-day-old seedlings after germination were growth in 13 cm deep circular plates filled with a 1:3 mixture of nutrient soil and vermiculite at 25°C under 16 h light/8 h dark in a growth room, and watered well during this period. After 12 days, water was stopped for 25 days, subsequently rehydration for 4 days, and then the survival rates were counted. In addition, after drought stress for 10 days, the fourth leaves from 2-week-old seedlings were collected for qRT-PCR analysis. For salt stress treatment, 14-day-old seedlings were subjected to the Yoshida’s culture solution containing 150 mM NaCl for 6 days at 25°C under 16 h light/8 h dark in a growth room, and subsequently grown in Yoshida’s culture solution. The survival rates were calculated after 10 days. After salinity stress for 2 days, the fourth leaves from 2-week-old seedlings were collected for qRT-PCR analysis. Each stress treatment experiment contained three biological replicates.

### Physiological and Biochemical Analysis of Transgenic Rice

After salinity stress for 3 days, the fourth leaves from 2-week-old seedlings were collected for measuring the relative electrolyte leakage (REL), proline and malondialdehyde (MDA) content, and catalase (CAT), and superoxide dismutase (SOD) activities. After drought stress for 15 days, the sixth leaves were used for measuring the electrolyte leakage, proline and MDA content, and CAT and SOD activities. REL and Proline content were detected based on the method of Tang et al. (2017). The MDA content from each samples was detected as previously described (Xia et al., 2018). The CAT and SOD activities were estimated according to a previously described method (Cai et al., 2015).

### RNA Isolation and qRT-PCR Analysis

Total RNA was isolated from rice different tissues using the Hipure Plant RNA Mini Kit (Magen^3^), and the DNase on Column Kit (Magen, see footnote 3) was used to digest DNA from each RNA sample following the manufacturer’s instructions. Subsequently, 4 μg of total RNA was used to synthesize first-strand cDNA using the PrimeScript^TM^ II 1st Strand cDNA Synthesis Kit (TAKARA, Beijing, China) based on the manufacturer’s instructions. LightCycler^®^ 480 Real Time PCR system (Roche, CA, United States) and TB Green^TM^ *Premix* Ex Taq II (Tli RNaseH Plus) (TAKARA, Beijing, China) were used for qRT-PCR analysis according to the manufacturer’s instructions. The 2^-ΔΔCT^ method was used to calculate the relative expression level, and *OsUbiquitin* was used as an internal reference gene for evaluating transcriptional abundance in rice. All qRT-PCR experiments described in the research contained three biological replicates, and each replicate had two technical replicates. All primer sequences used in this study were shown in Supplementary Table S1.

### Statistical Analysis

In this study, each experiment contained three biological replicates. Statistical analysis was performed using SAS software package according to the Duncan multiple range test (Duncan, 1955).

## Results

### Cloning and Bioinformatics Analysis of *OsMYB6* Gene

The full length open reading frame (ORF) of *OsMYB6* was cloned in pMD18-T vector and was confirmed by sequencing. The gene contained a 1,344 bp ORF encoding a putative protein of 447 amino acids, and its accession number in GenBank was MK061534. The predicted molecular weight of OsMYB6 protein was 46.3 KD, and with a calculated pI of 8.60.

**FIGURE 1:**
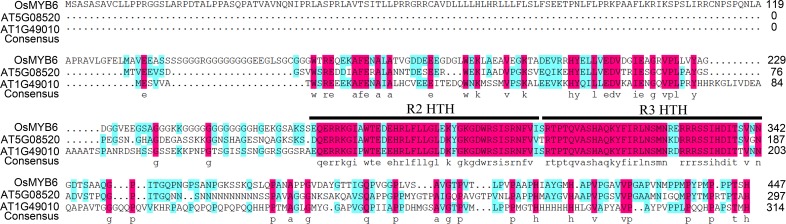
The amino acid sequence characteristics of OsMYB6 protein. The alignment of OsMYB6 and its ortholog proteins of *Arabidopsis*. R2 and R3 HTH represent the MYB repeat domain.

Amino acid sequence alignment analysis indicated that OsMYB6 was an R2R3-MYB protein that had two imperfect repeats in its MYB domain (Figure 1). In addition, the result further showed that OsMYB6 contained two typical structures for members of the R2R3-MYB gene family, namely R2-HTH (Helix Turn-Helix) and R3-HTH represented the MYB repeat domain (Figure 1), and these constituted its DNA-binding domain and were predict to bind the major groove of DNA.

### Expression Profile of *OsMYB6* Gene

**FIGURE 2:**
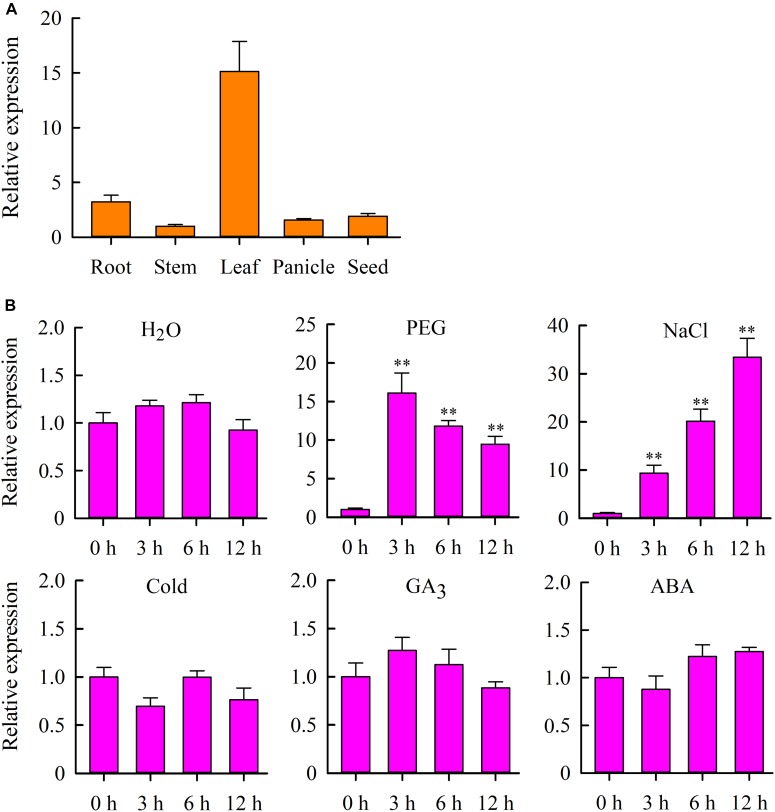
Expression analysis of the *OsMYB6* gene in rice. **(A)** qRT-PCR analysis of *OsMYB6* gene expression in various tissues. Bars show means ± SD of three biological replicates. **(B)** *OsMYB6* expression analysis (qRT-PCR) in leaves of 2-week-old rice seedlings subjected to H_2_O, 20% PEG 6000, NaCl, cold, ABA and GA_3_ treatments, respectively. Bars show standard deviations of the replicates. Each assay was run in triplicate for three independent biological replicates. Values represent means of *n* = 3 ± SD (Duncan test: ^∗∗^*P* < 0.01).

To gain more insight into the role of *OsMYB6* in plant growth and development, we analyzed the expression profile of *OsMYB6* gene in roots, stems, leaves, panicles and seeds using qRT-PCR. The results suggested that *OsMYB6* expression was detected in all organs tested, and this gene was highly expressed in leaves and low expressed in stems (Figure 2A).

We further examined the expression of *OsMYB6* gene in response to H_2_O, PEG, salinity, cold, GA_3_ and ABA stresses. The results indicated that PEG and salinity stresses induced the expression of *OsMYB6* at all treatment time points (Figure 2B). Under PEG stress conditions, the transcript level of *OsMYB6* was rapidly induced, and reached a peak within 3 h of treatment, and subsequent gene expression slowly decreased at 6–12 h. Under salinity stress, the expression of *OsMYB6* was also quickly induced, and showed a continuous rise after 3–12 h following the treatment, reaching a peak at 12 h. Under cold stress conditions, the expression of *OsMYB6* was slightly downregulated at 3 h, whereas no changes of the expression of *OsMYB6* were observed after 6–12 h following the treatment. Under GA_3_ treatment conditions, no changes in the *OsMYB6* transcript level was observed after 3–12 h following the treatment. Under ABA treatment conditions, the expression of *OsMYB6* was slightly downregulated at 3 h, and slightly up-regulated by ABA from 6 to 12 h. Under H_2_O treatment, no significant difference was found from 0 to 12 h. The observation of expression profile for *OsMYB6* indicated that *OsMYB6* gene might be very important for plant resistance against abiotic stress.

### *OsMYB6* Gene Encodes a Nuclear Localization Protein

**FIGURE 3:**
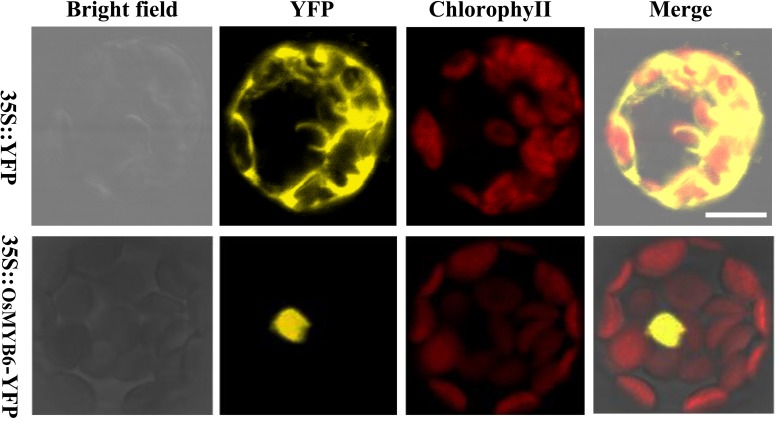
Subcellular localization of OsMYB6 protein. Cells were incubated with constructs carrying 35S::YFP or 35S::OsMYB6-YFP as described in “Materials and Methods” section. 35S::YFP and 35S::OsMYB6-YFP fusion proteins were transiently expressed under control of the CaMV 35S promoter in *Arabidopsis* protoplasts cells and observed with a laser scanning confocal microscope. Bar = 10 μm.

In order to detect the subcellular localization of OsMYB6 protein, the full-length coding sequence of *OsMYB6* without stop codon was cloned and the expression vector for OsMYB6-YFP (yellow fluorescent protein) fusion protein was constructed. Then the two vectors were transformed into *Arabidopsis* protoplasts cells, and the fluorescence signal was detected by using the laser scanning confocal microscopy. As shown in Figure 3, the YFP fluorescence signal from 35S:: YFP vector was detected throughout the whole cell, whereas the fluorescence of the 35S::OsMYB6-YFP construct was only detected in the nuclei. Taken together, our result suggested that *OsMYB6* gene encoded a nuclear localization protein.

### Phenotypic Analysis of Transgenic Rice With *OsMYB6* Gene

**FIGURE 4:**
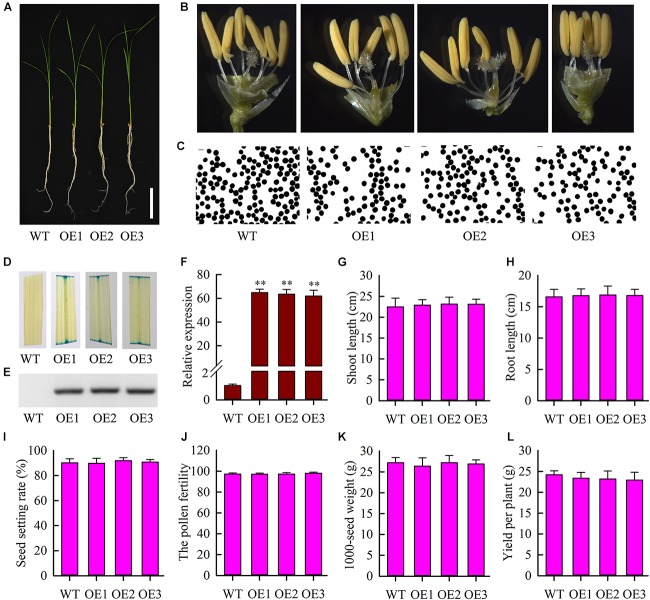
Phenotype of wild-type plants and transgenic plants with *OsMYB6*. **(A)** Phenotype of transgenic plants with *OsMYB6* and wild-type plants. Twelve-day-old seedlings cultured in Yoshida’s culture solution were photographed. Bar = 5 cm. **(B)** Flower structure analysis of the wild-type, OE1, OE2, and OE3 plants. **(C)** Pollen germination of the wild-type and transgenic plants. **(D)** GUS staining analysis in leaves from the wild-type, OE1, OE2, and OE3 plants. In addition, the pCAMBIA1301 vector contained a β-glucuronidase (GUS) gene under the control of the CaMV 35S promoter. **(E)** PCR analysis of the regenerated plants using gene specific primers of hygromycin gene. **(F)** qRT-PCR analysis of *OsMYB6* expression in the transgenic lines with *OsMYB6* and wild-type plants. OE1, OE2, and OE3 are three individual transgenic plants overexpressing *OsMYB6* gene. **(G)** Shoot length in transgenic plants overexpressing *OsMYB6* and wild-type plants after 12 days of growth on Yoshida’s culture solution. **(H)** Root length in transgenic plants overexpressing *OsMYB6* and wild-type plants after 12 days of growth on Yoshida’s culture solution. **(I)** Seed setting rate among the wild-type, OE1, OE2, and OE3 plants. **(J)** The pollen fertility from the wild-type, OE1, OE2, and OE3 plants. **(K)** 1000-grain weight comparison of the seeds from the wild-type, OE1, OE2 and OE3 plants. **(L)** Yield per plant among the wild-type, OE1, OE2, and OE3 plants. Values represent **(G–L)** means of *n* = 30 ± SD (Duncan test: ^∗∗^*P* < 0.01) from three independent biological experiments.

To further study the function of *OsMYB6* gene, we constructed overexpressing *OsMYB6* transgenic rice plants. Three independent T3 homozygous transgenic lines (OE1, OE2, and OE3) were selected for subsequent analysis using GUS staining, PCR and qRT-PCR. Phenotype analysis suggested that the growth, flower structure and pollen germination of transgenic plants with *OsMYB6* gene was similar to that of wild-type plants (Figure 4A–C). GUS staining showed that blue was detected in transgenic plant leaves (Figure 4D). Results from PCR analysis confirmed that hygromycin gene were integrated into the genome of transformed rice plants (Figure 4E). Expression analysis showed that *OsMYB6* expression in transgenic plants was significantly higher than in wild-type plants (Figure 4F). Furthermore, statistical analysis showed that compared to the wild-type plants, no significant difference was detected in the transgenic plants including shoot length, root length, seed setting rate, pollen fertility, 1000-seed weight and yield per plant (Figure 4G–L). Taken together, our results displayed that the overexpression of *OsMYB6* did not have a remarkable effect on the growth and development of rice.

### Overexpression of *OsMYB6* Increases Resistance of Transgenic Rice to Drought Stress

**FIGURE 5:**
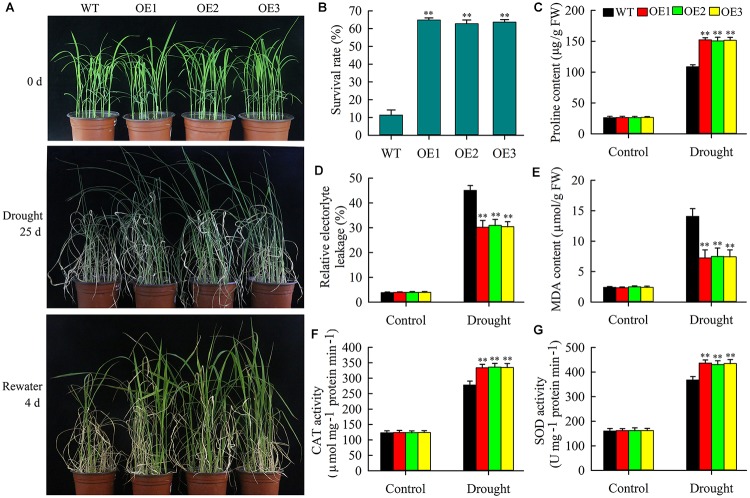
Drought stress analysis of wild-type plants and transgenic plants with *OsMYB6*. **(A)** Performance of 2-week-old seedlings from *OsMYB6* transgenic and wild-type plants subjected to drought stress without water for 25 days and then recovered for 4 days. Transgenic and wild-type plants were growth in 13 cm deep circular plates filled with a 1:3 mixture of nutrient soil and vermiculite at 25°C under 16 h light/8 h dark. The experiment contained three biological replicates. **(B)** Survival rates of transgenic and wild-type plants under drought stress. Values represent means of *n* = 3 ± SD (Duncan test: ^∗∗^*P* < 0.01) from three independent biological experiments. **(C)** Proline content in the wild-type and transgenic plants (OE1, OE2, and OE3) under normal growth and drought stress conditions. **(D)** The relative electrolyte leakage in the leaves of wild-type and *OsMYB6* transgenic plants under normal growth and drought stress conditions. **(E)** MDA content in the wild-type and transgenic plants under normal growth and drought stress conditions. **(F)** CAT activity in the wild-type and transgenic plants under normal growth and drought stress conditions. **(G)** SOD activity in the wild-type and transgenic plants under normal growth and drought stress conditions. Values represent **(C–G)** means of *n* = 30 ± SD (Duncan test: ^∗∗^*P* < 0.01) from three independent biological experiments.

As described above, *OsMYB6* expression was rapidly activated by PEG. Thus, we speculated that overexpression of *OsMYB6* might increase the resistance of transgenic plants to drought stress. To prove this result, the performance of 2-week-old wild-type and transgenic plant seedlings was explored in nutrient soil and vermiculite (1:3) under water deficiency conditions. As shown in Figure 5A, no significant morphological differences were observed between wild-type plants and transgenic plants with *OsMYB6* before drought stress treatment. However, after 25 days of drought stress, all wild-type plants suggested typical severe dehydration and significant wilting symptoms, and only a few leaves exhibited green color, whereas the leaves of most transgenic plants were still green. In addition, drought stress treatment also caused growth retardation of transgenic and wild-type plants but this effect was more pronounced for wild-type plants. Subsequently rehydration for 4 days, and then the survival rate was calculated. Our data showed that the survival rate of wild-type plants was 11.3%, which was significantly lower than that of transgenic plants (the survival rates of OE-1, 2, and 3 lines were 64.9%, 62.2%, and 63.7%, respectively) (Figure 5B).

Previous researches have indicated that plants suffering from drought and salt stresses often suggested accumulation of proline (Ben et al., 2012). Thus, we tested the proline content of the leaves from transgenic and wild-type plants under normal growth and drought stress conditions. The data displayed that no obvious difference in the content of proline between transgenic and wild-type plants under normal growth condition. However, compared with the wild-type plants, the transgenic plants showed significantly higher proline content after drought stress (Figure 5C). MDA and electrolyte leakage were closely correlated with the degree of cell membrane damage under abiotic stress (Mellacheruvu et al., 2016; James et al., 2018). Therefore, we further tested the REL and MDA content of the leaves from transgenic and wild-type plants. The results indicated that there was not significant differences were detected between transgenic and wild-type plants under well-irrigated conditions, whereas the wild-type plants suggested significantly higher REL and MDA content compared with the transgenic plants under drought stress conditions (Figure 5D,E).

Furthermore, we also examined the activities of CAT and SOD of the leaves from transgenic and wild-type plants under normal growth and drought stress conditions. The data indicated that after drought stress, both transgenic and wild-type plants exhibited increases in CAT and SOD activities, but compared with the wild-type lines, transgenic lines suggested a significantly higher CAT and SOD activities. Under normal growth conditions, there was no significant difference in the activities of CAT and SOD between *OsMYB6* overexpression and wild-type plants (Figure 5F,G). Collectively, our results show that overexpression of *OsMYB6* in rice can increase the resistance of transgenic plants to drought stress.

### Overexpression of *OsMYB6* Increases Resistance of Transgenic Rice to Salinity Stress

**FIGURE 6:**
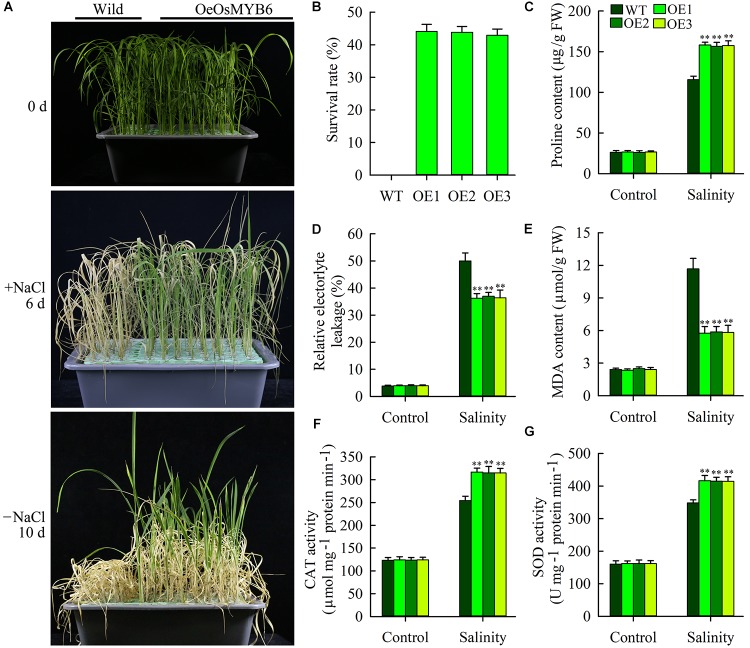
Salt stress tolerance of *OsMYB6* overexpression plants. **(A)** Performance of wild-type and transgenic plants before and after salt treatment (150 mM NaCl), and after recovery for 10 days following salt treatment. The experiment included three biological replicates. **(B)** Survival rates of wild-type and transgenic plants after recovery for 10 days following salt treatment. **(C)** Proline content of leaves before and after salt treatment. Values represent means of *n* = 30 ± SD from three independent experiments and the asterisks above the bars indicate significant differences from the corresponding wild-type (WT) at the *p* < 0.01 level. **(D,E)** Relative electrolyte leakage (REL) **(D)** and MDA content **(E)** of leaves before and after salt treatment. Values represent means of *n* = 30 ± SD from three independent experiments and the asterisks above the bars indicate significant differences from the corresponding WT at the *p* < 0.01 level. **(F,G)** Activity of catalase (CAT) **(F)** and superoxide dismutase (SOD) **(G)** in leaves before and after salt treatment. Values represent means of *n* = 30 ± SD from three independent experiments and the asterisks above the bars indicate significant differences from the corresponding WT at the *p* < 0.01 level.

In addition to PEG stress, NaCl stress also rapidly activated *OsMYB6* expression, showing that *OsMYB6* might be play an important role in response to salinity stress. Therefore, we further tested the resistance of wild-type and transgenic plants to salinity stress at the seedling stage. Two-week-old transgenic and wild-type seedlings were transferred to the Yoshida’s culture solution supplemented with 150 mM NaCl for 6 days. After 6 days, wild-type plants showed fewer green leaves, and more severe leaf rolling and wilting, whereas transgenic plants displayed more green leaves and less leaf wilting and rolling (Figure 6A). Subsequently, the wild-type and transgenic plants were moved to the Yoshida’s culture solution for 10 days. And then the survival rate was calculated. The data displayed that all seedlings from wild-type plants dead, whereas approximately 43.9% seedlings from transgenic plants survived and could regrow after the recovery (Figure 6B).

Changes in enzyme activities and physiological factors were also assessed in the leaves of transgenic and wild-type plants, including the REL, the contents of proline and MDA as well as the activities of CAT and SOD (Figure 6C–G). Under normal growth conditions, all the tested the activities of CAT and SOD and physiological factors were similar between the wild-type and transgenic plants. By contrast, the activities of CAT and SOD and physiological factors of both wild-type and transgenic plants were altered in response to salt stress. However, compared with the wild-type plants, the transgenic plants exhibited a lower REL, lower MDA content, higher proline content and higher CAT and SOD activities. Taken together, our results showed that overexpression of *OsMYB6* can also increase resistance of transgenic rice to salinity stress.

### Expression Analysis of Stress-Related Genes in Transgenic Plants and WT Plants Under Drought and Salinity Stresses

**FIGURE 7:**
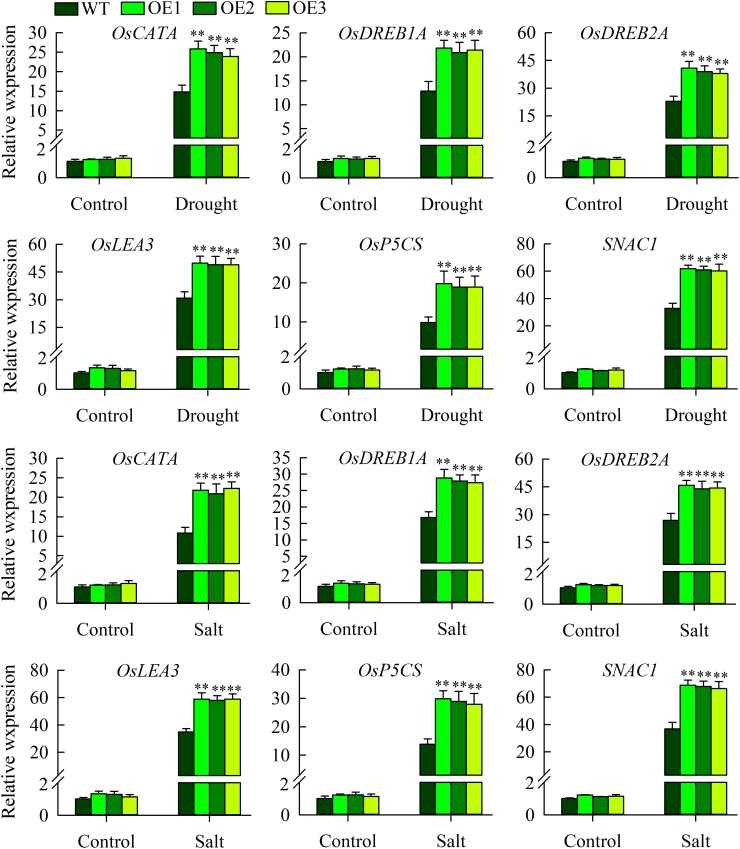
The expression of abiotic stress-responsive genes in the wild-type and transgenic plants under normal growth, drought and salt stress conditions. The experiment included three biological replicates, each with two technical replicates. Values represent means of *n* = 6 ± SD, and the asterisks above the bars indicate significant differences from the corresponding WT at the *p* < 0.01 level.

The above morphological experiments showed that *OsMYB6* transgenic plants had improved resistance to drought and salt stress. To elucidate the possible regulation mechanism underlying the increased drought and salinity tolerance observed in transgenic rice, we further tested the expression of abiotic-stress-related genes (*OsLEA3*, *OsDREB2A*, *OsDREB1A*, *OsP5CS*, *SNAC1*, *OsCATA*) under normal growth and drought and salinity stress conditions using qRT-PCR, since overexpression of these genes can increase the tolerance of transgenic plants to abiotic stress (Dubouzet et al., 2003; Hu et al., 2006; Hu, 2008; Mallikarjuna et al., 2011; Liu G. et al., 2014). Our results displayed that compared to under normal growth conditions, the expression of *OsLEA3*, *OsDREB2A*, *OsDREB1A*, *OsP5CS*, *SNAC1*, and *OsCATA* was constitutively elevated in the transgenic and wild-type plants under drought and salinity stress conditions (Figure 7). However, the expression of these genes were significantly lower in the wild-type plants than in the transgenic plants after drought and salinity stress treatment. In addition, there was no significant difference in the expression of these abiotic stress-related genes between transgenic plants and wild-type plants under normal growth conditions (Figure 7).

## Discussion

The MYB proteins contain quite a large family, with 183 members in rice (Chen et al., 2006), and meaningful progress has been made in understanding the biological function of the *MYB* genes (Stracke et al., 2001; Liu C. et al., 2014; Zhu et al., 2015). Although some rice MYB family genes, such as *OsMYB30* (Lv et al., 2017), *OsARM1* (Wang et al., 2017), *OsMYB2* (Yang et al., 2012), *OsMYB91* (Zhu et al., 2015), and *OsMYB1* (Gu et al., 2017), have been demonstrated to play an important role in plant growth, development and abiotic stress response, the biological function of most *OsMYB* genes remains unclear and needs further study. In this study, we report the isolation and characterization of the *OsMYB6*, a member of the rice MYB family. Our data suggest that *OsMYB6* is a stress response gene and that, when overexpressed in rice, it does not affect the growth and development of transgenic rice, but improves the resistance of transgenic rice to drought and salt stress.

Increasing evidence have demonstrated that some *MYB* genes are induced or inhibited expression by abiotic stress, and overexpression of these genes can increase the tolerance of transgenic plants to drought or salt stress (Yang et al., 2012; Wei et al., 2017). Similarly, our results displayed that drought and salinity stress could rapidly activate *OsMYB6* gene expression (Figure 2B), suggesting *OsMYB6* may play critical roles in drought and salinity stress. Therefore, we constructed *OsMYB6* overexpressing transgenic plants and tested their resistance to drought and salinity stress. Our finding suggested that overexpression of *OsMYB6* in rice increased transgenic plants tolerance to drought and salinity stress, and transgenic plants possessed less pronounced leaf rolling and aging compared to wild-type plants (Figure 5A, 6A). When plants are subjected to abiotic stress such as drought and salinity, some physiological indices (REL, proline, and MDA) can react quickly to enable these plants to survive under these extreme environmental conditions (Tang et al., 2017; Wei et al., 2017; James et al., 2018). Thus, physiological indices related to the plants’ osmotic stress caused by drought and salinity can be used as a fast and accurate method for assessing plant resistance to abiotic stress. REL, an important parameter to reflect the degree of cell membrane damage (James et al., 2018), was higher in the leaves from wild-type plants than in the leaves from transgenic plants (Figure 5D, 6D), suggesting that the *OsMYB6* gene may protect cell membrane integrity of plants in response to drought and salinity stress. Abiotic stress has been reported to cause lipid peroxidation, leading to MDA accumulation (Mellacheruvu et al., 2016; James et al., 2018). Our data displayed that the MDA content was higher from wild-type plants than from transgenic plants with *OsMYB6* (Figure 5E, 6E), which shows that drought and salinity stress have more damage to wild-type plants compared to transgenic plants. It has been reported that proline acts as a stabilizer to protect cells against abiotic stress (Ben et al., 2012). In this study, the transgenic plants accumulated more proline compared with the wild-type plants under drought and salinity stress (Figure 5C, 6C), suggesting that proline may be a factor responsible for the higher tolerance to drought and salinity stress shown by the transgenic plants overexpression *OsMYB6*. Furthermore, SOD and CAT have been reported to be vital antioxidant enzymes that protect plants from abiotic stress damage (Sofo et al., 2015; Del Río et al., 2018). In this work, the transgenic plants possessed higher CAT and SOD activities compared to the wild-type plants (Figure 5F,G, 6F,G), further providing evidence to support this result regarding transgenic plants increase drought and salinity stress resistance. Collectively, these results demonstrated that the increased drought and salinity stress tolerance of the transgenic plants with *OsMYB6* was at least partially related to lower MDA content, lower REL, higher proline content and higher CAT and SOD activities.

In addition to the physiological indicators and enzymatic activities described above, the increase tolerance to drought and salinity stress also attribute to the significantly induce expression of abiotic stress-response genes (Mao et al., 2012; Zhu et al., 2015; Gu et al., 2017). For example, overexpression of *TaNAC2* results in enhance tolerances to drought, salt and freezing stress by enhancing expression of abiotic stress-response genes (Mao et al., 2012). Similarly, in our study, some abiotic stress-response genes, including *OsLEA3*, *OsDREB2A*, *OsDREB1A*, *OsP5CS*, *SNAC1*, *OsCATA*, were more highly expressed in transgenic plants than in wild-type plants under drought and salinity stress (Figure 7). The *OsCATA* gene products are involved in protecting plants from damage caused by reactive oxygen species (Sirko et al., 2003). It has been demonstrated that there is a positive correlation between the expression of *OsP5CS* gene and the accumulation of proline, and overexpression of *OsP5CS* increases resistance to abiotic stress in transgenic plants (Hien et al., 2003). Subsequent studies show that *OsLEA3*, *OsDREB2A*, *OsDREB1A*, and *SNAC1* are stress-inducible genes that improve transgenic plants’ tolerance to abiotic stress (Dubouzet et al., 2003; Hu et al., 2006; Hu, 2008; Mallikarjuna et al., 2011; Liu G. et al., 2014). However, no significant difference in the expression of these abiotic stress-related genes was found between the *OsMYB6*-overexpressing and wild-type plants under normal growth conditions (Figure 7), despite the fact that the constitutive promoter was used to drive the gene. One possible explanation is that other stress-responsive regulators are required to activate *OsMYB6*-dependent, stress-responsive genes under stressed conditions. A similar observation has been reported in *nNOS*-overexpressing lines (Cai et al., 2015). For example, overexpression of *nNOS* in rice enhances the expression of stress-responsive genes under abiotic stress, but both wild-type and transgenic lines exhibited similar expression levels of all the tested genes under normal conditions. Taken together, the results indicate that the transgenic plants have more resistance to drought and salinity stress, possibly by enhancing the expression of these abiotic stress-response genes in response to drought and salinity stress. In summary, these results support the conclusion that *OsMYB6* encodes a stress-responsive MYB transcription factor that plays a regulatory role in tolerance of rice to salt and drought stress. More importantly, overexpression of *OsMYB6* in rice seedlings did not affect their phenotypes under normal growth conditions. Therefore, *OsMYB6* provides a promising tool for improving the tolerance of rice to abiotic stress in general and to drought and salt stress in particular.

## Conclusion

In this study, we isolated and characterized the rice *OsMYB6* gene, which was induced by drought and salinity stress. Based on the performance of transgenic plants under abiotic stress, we conclude that the *OsMYB6* gene suggests critical functions in improving plant tolerance to drought and salinity stress. These results enhance our understanding of the role of rice MYB transcription factor in the regulation of abiotic stress response, and provide a candidate gene for the cultivation of drought-tolerant and salt-tolerant rice varieties.

## Author Contributions

YZ, YG, QWu, XY, LZ, JL, and JZ carried out the experiments. WH, WL, QWa, CJ, and ZL analyzed the data. YT and KL conceived and designed the research. YT and XB wrote and revised the manuscript. All authors read and approved the final manuscript.

## Conflict of Interest Statement

The authors declare that the research was conducted in the absence of any commercial or financial relationships that could be construed as a potential conflict of interest.

## Footnotes

1 https://www.ncbi.nlm.nih.gov/

2 https://web.expasy.org/protparam/

3 http://www.magentec.com.cn/
